# Do Cognitive–Achievement Relations Vary by General Ability Level?

**DOI:** 10.3390/jintelligence11090177

**Published:** 2023-09-04

**Authors:** Daniel B. Hajovsky, Christopher R. Niileksela, Sunny C. Olsen, Morgan K. Sekula

**Affiliations:** 1 Department of Educational Psychology, Texas A&M University, College Station, TX 77845, USA; 2 Department of Educational Psychology, University of Kansas, Lawrence, KS 66045, USA

**Keywords:** cognitive ability, academic achievement, Woodcock–Johnson, cognitive–achievement relations, Spearman’s Law of Diminishing Returns

## Abstract

Cognitive–achievement relations research has been instrumental in understanding the development of academic skills and learning difficulties. Most cognitive–achievement relations research has been conducted with large samples and represent average relations across the ability spectrum. A notable gap in the literature is whether these relations vary by cognitive ability levels (IQ). This study examined cognitive–achievement relations across different general ability levels (Low, Average, and High) to fill this gap. Based on Spearman’s Law of Diminishing Returns, it would be expected that general intelligence would be a stronger predictor of academic skills at lower levels of IQ, and more specific abilities would be stronger predictors of academic skills at higher levels of IQ. To test this, multi-group path analysis and structural equation modeling were used to examine whether integrated models of cognitive–reading relations are differentiated by IQ levels in the Woodcock–Johnson III and Woodcock–Johnson IV standardization samples. Global and broad cognitive abilities were used as predictors of basic reading skills and reading comprehension for elementary and secondary school students. The magnitude of prediction differed across ability groups in some cases, but not all. Importantly, the variance explained in basic reading skills and reading comprehension tended to be larger for the Low group compared to the Average and High groups. When variance accounted for by general intelligence was removed from the broad abilities, the effects of the broad abilities were similar across ability groups, but the indirect effects of *g* were higher for the Low group. Additionally, *g* had stronger relative effects on reading in the Low group, and broad abilities had stronger relative effects on reading in the Average and High groups. The implications and limitations of this study are discussed.

## 1. Introduction

It is well known that individual differences in human cognitive functioning are associated with variation in educational outcomes (Gottfredson 1997; Jensen 1998; Kaufman et al. 2012; Rosén et al. 2017). These associations, often called cognitive–achievement relations in the literature, have been found across different cognitive ability domains and reading, writing, and mathematics achievement (Caemmerer et al. 2018; Hajovsky et al. 2018; Niileksela et al. 2016). Standardized test scores from cognitive ability and academic achievement measures are frequently used within high-stakes decision making, especially specific learning disability (SLD) evaluations (Maki et al. 2015). Understanding variation in the associations between global and broad cognitive abilities and different domains of academic achievement is paramount to understanding learning difficulties and informing assessment practices. 

A notable gap in the literature is whether cognitive–achievement relations observed in previous research generalize to different cognitive ability levels (e.g., higher/lower IQ). On average, students with learning difficulties often have slightly lower levels of cognitive abilities (Johnson et al. 2010), possibly due to specific cognitive deficits that are related to their learning challenges (Grigorenko et al. 2020). Much of the research on cognitive–achievement relations uses standardization samples from norm-referenced test batteries (Caemmerer et al. 2018; Hajovsky et al. 2018; Niileksela et al. 2016). The findings from this research represent relations across the ability spectrum, but an assumption of those findings is that the magnitude of relations generalizes across the ability spectrum. If relations among cognitive abilities and academic skills differ by ability level, and those with SLD tend to have slightly lower cognitive abilities, then the research on cognitive–achievement relations may not generalize to students with SLD. The purpose of this study is to examine cognitive–achievement relations across different general ability levels in school-age children to determine if these generalize across the IQ distribution. 

## 2. Spearman’s Law of Diminishing Returns (SLODR)

A potential reason that differences in cognitive–achievement relations may exist across ability level may be understood through the lens of Spearman’s Law of Diminishing Returns (SLODR; Spearman 1927). Correlations among tests are typically higher for individuals with lower levels of general intelligence (*g*) compared to those with higher levels of *g*, suggesting greater differentiation among broad abilities (e.g., visual processing, working memory) for those with higher IQ scores (Abad et al. 2003; Detterman and Daniel 1989). According to the theory of cognitive ability differentiation (CAD; Jensen 2003), *g* contributes less to broad abilities when *g* is higher, resulting in more differentiation among broad abilities (weaker correlations). Thus, CAD posits that the variance in broad abilities not explained by *g* (residual variance or group factors) will be larger in the higher *g* group. Conversely, *g* contributes more to broad abilities when *g* is lower, resulting in less differentiation among broad abilities (stronger correlations), which reflects more underlying variance shared by the broad abilities that can be attributed to *g* (Reynolds et al. 2011). 

A potential implication underlying this theoretical proposition is that the relations between *g* (or global IQ) and academic achievement will be stronger at lower levels of *g*, and the relations between broad cognitive abilities (e.g., working memory) and academic achievement will be stronger at higher levels of *g* (McLarnon et al. 2018; Murray et al. 2013). It is unknown to what extent cognitive–achievement relations are differentiated by IQ level. Thus, we aim to test whether cognitive–achievement relations vary by level of IQ (a strong proxy of *g*; Reynolds et al. 2013) using two different standardized cognitive and achievement batteries co-normed within nationally representative samples.

## 3. Cognitive–Achievement Relations

Most of the prior cognitive–achievement relations research has been completed with large samples representing average relations across the ability spectrum. Research supports the moderation of cognitive–achievement relations by development (e.g., Caemmerer et al. 2018; Floyd et al. 2012; Hajovsky et al. 2014; Niileksela et al. 2016), gender (e.g., Hajovsky et al. 2018), and mixed results with regard to race/ethnicity (e.g., Hajovsky and Chesnut 2022; Keith 1999; Weiss and Prifitera 1995). Moreover, the theory of mutualism expands on the cognitive–academic bidirectional relationship (Peng and Kievit 2020). In cognitive–academic mutualism, exposure to broader educational resources increases both academic achievement and cognitive performance for students with high abilities, leading to stronger relationships between cognitive ability and academic achievement. As students progress through each successive grade level, the mutualistic effects become more pronounced with high academic and cognitive ability levels (Zhang and Peng 2023). In other words, the theory of mutualism suggests that the development of cognitive ability and academic achievement is bidirectional and that they have influences on each other, in contrast to a unidirectional relationship where cognitive ability only influences academic achievement, but not vice versa. Furthermore, this theory hypothesizes that the relation between relevant, specific cognitive abilities (e.g., reasoning, working memory) and reading/mathematics achievement should increase with age as people develop their skills in these areas (Peng and Kievit 2020). While mutualism between verbal working memory and academic skills is mixed, it is suggested that one reason for inconclusive findings may be due to a lack of analyses that account for moderating variables (i.e., ability level). Peng and Kievit (2020) have hypothesized that individuals with high abilities create more mutualistic skills (i.e., cognition and academic skills).

While studying cognitive–achievement relations has been well documented, there is a recent push for integrated models in the literature (e.g., Hajovsky et al. 2014; Niileksela et al. 2016; Feraco et al. 2022). Integrated models of cognitive–achievement relations suggest that cognitive abilities influence more advanced academic skills via basic academic skills. For example, general and broad cognitive abilities influence reading comprehension via basic reading skills (e.g., Floyd et al. 2012; Hajovsky et al. 2014) and math problem solving via math computation skills (e.g., Decker and Roberts 2015). However, this research base focuses exclusively on the average relations between cognitive and achievement scores without consideration of whether associations vary at different ability or achievement levels. Research employing quantile regression has examined cognitive–achievement relations as a function of achievement level (e.g., Language and Reading Research Consortium and Logan 2017). These findings suggest cognitive–achievement relations vary by academic skill level (as a function of the outcome variable or achievement), but this work does not address whether associations vary at different ability levels (as a function of the predictor variable or IQ). 

There has been limited empirical work exploring differentiation of cognitive–achievement relations by *g*. In one notable study, McLarnon et al. (2018) examined how global and narrow cognitive measures derived from the Medical College Admission Test (MCAT) predicted GPA in high-*g* versus low-*g* individuals. They found *g* was a stronger predictor of GPA in the low-*g* versus the high-*g* group, providing support for differentiation of cognitive ability across the ability spectrum at the global cognitive level. Although it was predicted that narrow cognitive measures derived from the MCAT would be a stronger predictor of GPA in the higher-*g* group (due to larger residual variances), it was found that the low-*g* group showed stronger relations between narrow cognitive measures and GPA (McLarnon et al. 2018). In a more recent study using the standardization data for an IQ test in a German sample, researchers found that broad cognitive abilities had minimal incremental prediction on school grades in the low-IQ and mid-IQ groups but had a significantly stronger effect on school grades in the high-IQ group after accounting for a *g*-factor score (Breit and Preckel 2020). These mixed findings suggest some evidence of differentiation of cognitive–achievement relations by *g*, but this phenomenon has not been examined within U.S. nationally representative school-age samples utilizing standardized measures of cognitive ability and academic achievement.

## 4. Current Study

Cognitive–achievement relations research has been instrumental in understanding learning difficulties and in the development of neurocognitive models of assessment (Alfonso and Flanagan 2018; Schneider and Kaufman 2017). Most of this research is completed with large samples representing average relations across the ability spectrum, but it is possible that relations found in previous research do not generalize to individuals with higher or lower general intelligence (IQ). Nonetheless, the cognitive–achievement relations studies used to inform diagnostic models have rarely quantified cognitive–achievement relations by IQ level (e.g., low, average, or high). This is a non-trivial consideration as the interpretive weight attributed to basic psychological processes (e.g., phonological processing) for understanding academic functioning may shift as a function of IQ level. If the relations among global and broad cognitive abilities and academic skills differ by ability level, then the research on cognitive–achievement relations may not apply to students with suspected SLD or lower cognitive functioning. 

To address this gap in the literature, we use multi-group structural equation modeling to examine whether integrated models of cognitive–achievement relations are differentiated by different IQ levels for school-age children and adolescents. A benefit of this study is the use of two large, nationally representative samples to examine the differentiation of cognitive–achievement relations by IQ level. One consideration in a comparison of different IQ groups based on a selection of cut-points using a variable that is included in the model (i.e., a general ability composite) is a concern with restriction of range related to dichotomizing a continuous variable (Reynolds et al. 2010). To mitigate this issue, we developed an alternative general ability composite using test scores that are not included in any of the general or broad ability composites used in the models we tested for differentiation. This alternative general ability composite was used to create groups. We use composite scores in the analyses as these scores are utilized in diagnostic assessment decision making and create Low (<25th percentile), Average (25th–75th percentile), and High (>75th percentile) ability groups. We then examine integrated models of cognitive–achievement relations in each of these groups to determine if they are similar or different, with a focus on both global IQ-achievement relations and broad cognitive ability–achievement relations for basic reading skills and reading comprehension. 

This study aims to test two general hypotheses: (a) general ability (IQ) and basic reading skills and reading comprehension relations will be stronger in the Low group relative to the Average or High groups; and (b) broad cognitive ability and basic reading skills and reading comprehension relations will be stronger in the High group relative to the Low and Average groups.

## 5. Method

### 5.1. Participants

The normative samples for the Woodcock–Johnson Third Edition (WJ III) (Woodcock et al. 2001, 2007) and Woodcock–Johnson Fourth Edition (WJ IV) Tests of Cognitive Abilities and Achievement were used for this study (Schrank et al. 2014a, 2014b). Both samples were used to replicate the findings across two different samples and test batteries. The WJ III and WJ IV standardization samples are nationally representative samples of children, adolescents, and adults ages 2 to 90+ years. The WJ III normative sample had 8782 individuals, with 4470 individuals in kindergarten through 12th grade. The WJ IV normative sample had 7416 individuals, with 3891 individuals in kindergarten through 12th grade. Participants in both samples were stratified based on age by the following demographic variables: race/ethnicity, sex, country of birth, community type, U.S. census region, parent education, school type, college type, occupational level, and employment status (McGrew et al. 2014, 2007). For this study, the WJ III and WJ IV samples were split into elementary (1st through 5th grade) and secondary (6th through 12th grade) samples. The samples were split into elementary and secondary samples because previous research has suggested that age moderates the relations between cognitive abilities and academic skills (e.g., Floyd et al. 2012; Niileksela et al. 2016). Average ages for grade levels across the elementary sample ranged from 6.5 years in 1st grade to 10.5 years in 5th grade. Average ages for grade levels across the secondary sample ranged from 11.5 years to 17.5 years.

### 5.2. Measures

The WJ III and WJ IV provide several composites for measuring intellectual and achievement abilities according to the Cattell–Horn–Carroll theory (CHC; Schneider and McGrew 2018). From both batteries, corresponding composites for general intelligence, the General Intellectual Ability (GIA), the seven broad CHC abilities (G*c*, G*f*, G*sm*, G*s*, G*lr*, G*v*, G*a*), and two reading composites including Basic Reading Skills and Reading Comprehension were used in this study. The WJ III Technical Manual (McGrew and Woodcock 2001) contains extensive validity information of the measures guided by CHC theory (Schneider and McGrew 2018). The WJ IV Technical Manual provides extensive concurrent, criterion, and developmental validity evidence that includes data on patterns of intercorrelations among tests and clusters and a three-stage structural validity analysis using factor analysis, cluster analysis, and multidimensional scaling (McGrew et al. 2014).

### 5.3. Basic Reading Skills 

The Basic Reading Skills (BRS) composite provides a measure of an individual’s reading ability in English word identification and phonetic abilities. On both the WJ III and WJ IV, the composite includes the Letter–Word Identification and Word Attack subtests, where examinees read single real words and nonsense words, respectively. The WJ III Basic Reading Skills cluster reliability coefficients ranged from 0.90 to 0.98 across ages 5–19 (McGrew and Woodcock 2001; McGrew et al. 2007). The WJ IV Basic Reading Skills cluster reliability coefficients ranged from 0.93 to 0.98 across ages 5–19 (McGrew et al. 2014). 

### 5.4. Reading Comprehension 

The Reading Comprehension (RC) composite measures an individual’s understanding of what they have read. On the WJ III, the composite includes Passage Comprehension, where examinees supply words to fill in missing blanks in a sentence or paragraph, and Reading Vocabulary, where examinees supply synonyms and antonyms of words they read. The subtests included in this composite differ slightly on the WJ IV. It still includes Passage Comprehension, but instead of Reading Vocabulary, it includes Reading Recall, where examinees read a story silently and retell the story from memory. The WJ III Reading Comprehension cluster reliability coefficients ranged from 0.88 to 0.97 across ages 5–19 (McGrew and Woodcock 2001; McGrew et al. 2007). The WJ IV Reading Comprehension cluster reliability coefficients ranged from 0.91 to 0.99 across ages 5–19 (McGrew et al. 2014). 

### 5.5. General Intellectual Ability

The General Intellectual Ability (GIA) composite provides a snapshot of an individual’s current intellectual functioning and is representative of *g* in CHC theory. The global intelligence measure includes one subtest representing each of the seven CHC broad abilities measured by both WJ batteries. The subtests differ on the WJ III and WJ IV. The WJ III uses scores from Verbal Comprehension, Concept Formation, Sound Blending, Spatial Relations, Visual–Auditory Learning, Visual Matching, and Numbers Reversed. The WJ IV uses scores from Oral Comprehension, Number Series, Verbal Attention, Letter–Pattern Matching, Phonological Processing, Story Recall, and Visualization. The WJ III GIA cluster reliability coefficients ranged from 0.96 to 0.97 across ages 5–19 (McGrew and Woodcock 2001; McGrew et al. 2007). The WJ IV GIA cluster reliability coefficients ranged from 0.95 to 0.97 across ages 5–19 (McGrew et al. 2014). 

### 5.6. Broad CHC Cognitive Abilities

Composite scores for the seven broad CHC abilities were used from the WJ III and WJ IV. These broad abilities are measured by two subtests, each of which measures a different narrow ability that is subsumed under the broad ability. The tests used on the broad CHC composites differ slightly across the WJ III and WJ IV but reflect the same underlying construct across measures. We provide a definition from the WJ IV, which includes: 

Comprehension–Knowledge (G*c*). This measures the depth and breadth of declarative and procedural knowledge and skills valued by one’s culture. It is measured by Verbal Comprehension and General Information on the WJ III, and Oral Comprehension and General information on the WJ IV.

Fluid reasoning (G*f*). This measures the deliberate and controlled focused attention to solve novel problems that cannot be solved using prior knowledge. It is measured by Concept Formation and Analysis–Synthesis on the WJ III, and Number Series and Concept Formation on the WJ IV.

Visual processing (G*v*). This measures the ability to use mental imagery, store images in primary memory, or perform visual–spatial analysis or mental transformation of images. It is measured by Spatial Relations and Picture Recognition on the WJ III, and Visualization and Picture Recognition on the WJ IV. 

Short-term working memory (G*wm*). This measures the ability to encode, maintain, and/or manipulate auditory or visual information in primary memory to solve multiple-step problems. It is measured by Numbers Reversed and Memory for Words on the WJ III, and Verbal Attention and Numbers Reversed on the WJ IV.

Auditory processing (G*a*). This measures the ability to perceive, discriminate, and manipulate sound information, including processing of auditory information in primary memory and activation, restructuring, or retrieval of information from semantic–lexical memory. It is measured by Sound Blending and Auditory Attention on the WJ III, and Phonological Processing and Nonword Repetition on the WJ IV.

Cognitive processing speed (G*s*). This measures the ability to control attention to automatically and fluently perform relatively simple repetitive cognitive tasks. It is measured by Visual Matching and Decision Speed on the WJ III, and Letter–Pattern Matching and Pair Cancellation on the WJ IV.

Long-term retrieval (G*lr*). This measures the ability to store information and fluently retrieve it later (Schneider and McGrew 2018). It is measured by Visual–Auditory Learning and Retrieval Fluency on the WJ III, and Story Recall and Visual–Auditory Learning on the WJ IV.

The WJ III broad ability cluster reliability coefficients varied from 0.86 to 0.96 across ages 5–19, except G*v*, which ranged from 0.70 to 0.84, and G*sm*, which ranged from 0.83 to 0.91 (McGrew and Woodcock 2001; McGrew et al. 2007). The WJ IV broad ability cluster reliability coefficients varied from 0.88 to 0.98 across ages 5–19, except G*v*, which ranged from 0.80 to 0.89 (McGrew et al. 2014). 

## 6. Data Analytic Plan

Observed scores were used in all analyses. These were chosen for two reasons. First, in most cases, there would only be two tests available for each broad CHC ability factor because several of the extra tests that could be used as indicators in a latent variable model were used to create the alternative GIA composite used to select ability groups. Second, previous research with the WJ III and WJ IV has often had difficulties appropriately estimating latent variable models, such as having second-order factor loadings that are equal to or greater than one (e.g., Floyd et al. 2012; Niileksela et al. 2016).

### 6.1. Identifying Ability Groups

Groups representing Low (<25th percentile), Average (25th–75th percentile), and High (>75th percentile) ability were selected from the WJ III and WJ IV normative samples. These percentiles were used to define groups for two primary reasons. First, these values were used to ensure adequate power and sample sizes. Setting these values at lower and higher percentiles (e.g., +/− 1 standard deviations, or 16th and 84th percentiles) would have resulted in substantial differences in sample sizes across ability groups and would have reduced power. Even when set at the 25th and 75th percentiles, the sample size of the Average ability group was twice as large as the Low and High ability groups. Second, the 25th percentile has been suggested as a point at which some cognitive or academic skills may be considered as requiring further attention in evaluations (e.g., Fletcher et al. 2019), so there is also a practical precedent for using these values to select groups, especially the Low group. 

It is problematic to select groups based on the variables that will be used in the analysis because this attenuates the distribution of scores and results in a restriction of range. To avoid this issue, ability groups were selected using an alternative estimate of general intellectual ability (altGIA). This altGIA was estimated using subtests from the WJ III and WJ IV that were not included in the GIA or the broad CHC composites. None of the subtests used to identify the groups were included in composite scores used in any subsequent analyses. This approach accounts for the statistical issues that arise when performing analyses with the variables that were used to select groups.

Seven tests that represent the seven broad CHC abilities on the WJ III were selected to estimate the altGIA, including Picture Vocabulary (G*c*), Memory for Names (G*lr*), Block Rotation (G*v*), Incomplete Words (G*a*), Number Series (G*f*), Cross Out (G*s*), and Memory for Sentences (G*sm*). A one-factor model was created using these tests, and factor scores were estimated for each individual in the sample. Those scores were then used to select the Low (<25th percentile), Average (25th–75th percentile), and High (>75th percentile) ability groups. The one-factor model and factor scores were estimated separately for the elementary and secondary samples. The validity of the altGIA was established by correlating the latent factor of the altGIA with the latent factor of the tests included in the GIA on the WJ III. The correlation between the latent *g* for the WJ III GIA and altGIA was .99 for both the elementary and secondary samples, suggesting they were essentially equivalent at the latent level. The coefficient omega for the altGIA was .69 for the elementary sample and .74 for the secondary sample. For comparison, the coefficient omega for the WJ III GIA was .77 for the elementary sample and .81 for the secondary sample. Although omega values for the altGIA were slightly lower than the GIA, these values still suggest adequate reliability of the altGIA on the WJ III.

Seven tests that represent the seven broad CHC abilities on the WJ IV were selected to estimate the altGIA: Picture Vocabulary (G*c*), Analysis Synthesis (G*f*), Number Pattern Matching (G*s*), Memory for Words (G*sm*), Sound Blending (G*a*), Memory for Names (G*lr*), and Visual Closure (G*v*). Similar to the WJ III procedure, a one-factor model was created using these tests, and factor scores were estimated for each individual in the sample; those scores were used to select the three ability groups separately for the elementary and secondary samples. The validity of the altGIA was established by correlating the latent factor of the altGIA with the latent factor using the general factor by using the tests included in the GIA used on the WJ IV. The correlation between the latent *g* for the WJ IV GIA and altGIA was 1.00 for both the elementary and secondary samples, suggesting that they were essentially equivalent at the latent level. The coefficient omega for the altGIA was .69 for the elementary sample and .70 for the secondary sample. For comparison, the coefficient omega for the WJ IV GIA was .82 for the elementary sample and .81 for the secondary sample. Although omega values for the altGIA were slightly lower than the GIA, these values still suggest adequate reliability of the altGIA on the WJ IV.

### 6.2. Integrated Cognitive–Achievement Models

All models used methods of multi-group path analysis and structural equation modeling (MG-SEM). In this approach, a single model is estimated simultaneously across groups; in this case, the Low, Average, and High ability groups. Cross-group equality constraints are then added to the model to determine if there are statistically significant differences across groups on specific model parameters. In this study, the equality of regression paths between cognitive abilities and reading skills was of primary interest. The likelihood ratio test was used to test nested models (i.e., models with cross-group equality constraints were compared to models without cross-group equality constraints). A statistically significant degradation in model fit would suggest that the paths are not statistically equal across groups, and a non-statistically significant degradation in model fit would suggest that paths are statistically equal across groups.

Three sets of models were planned for this study. An integrated model of cognitive–achievement relations was estimated for each of the three models, where cognitive abilities predicted both BRS and RC, and the BRS predicted RC (e.g., there were only direct effects of cognitive abilities on BRS, but there were direct effects of cognitive abilities on RC and indirect effects of cognitive abilities on RC through BRS). 

**Figure 1:**
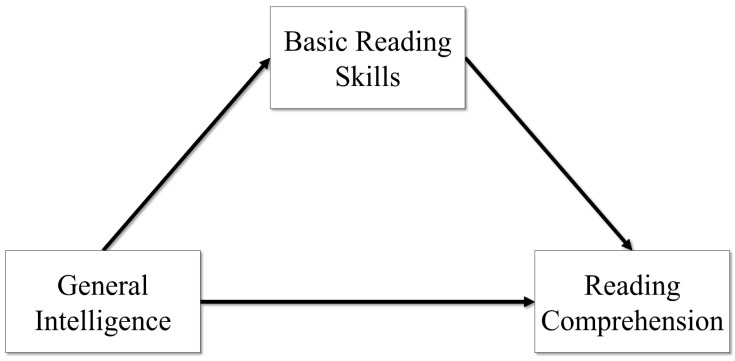
General intelligence predicting reading.

First, a model was estimated where the GIA predicted both BRS and RC, and BRS predicted RC. This was a simple mediation model that assumes the GIA is a predictor of both BRS and RC, and then BRS also predicts RC, where the effects of the GIA on RC may be partially mediated through BRS. This model only considers the effects of general intelligence on reading skills. The model is depicted in Figure 1.

**Figure 2:**
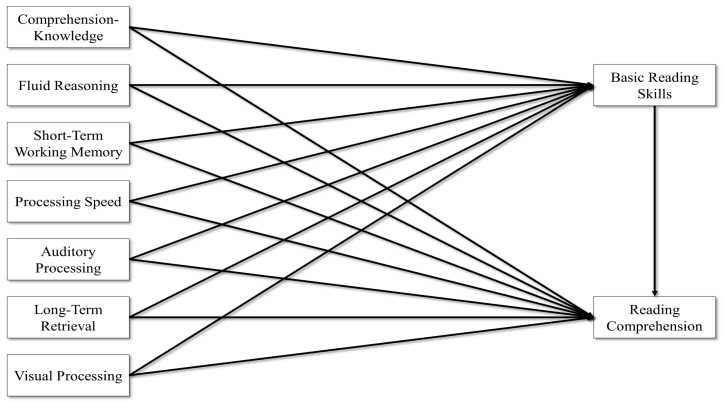
Broad CHC abilities predicting reading.

Second, a model where the seven broad CHC abilities predicted reading was estimated. In this model, the seven broad CHC abilities predicted both BRS and RC, and then BRS predicted RC. Like the first model, this model assumes that the broad CHC abilities predict both BRS and RC, and BRS also predicts RC, where the effects of the broad CHC abilities on RC may be partially mediated through BRS. The model is depicted in Figure 2. This model examines the effects of the broad CHC abilities on reading skills but does not necessarily partial out variance that can be attributed to general intelligence and to the specific broad CHC abilities. 

**Figure 3:**
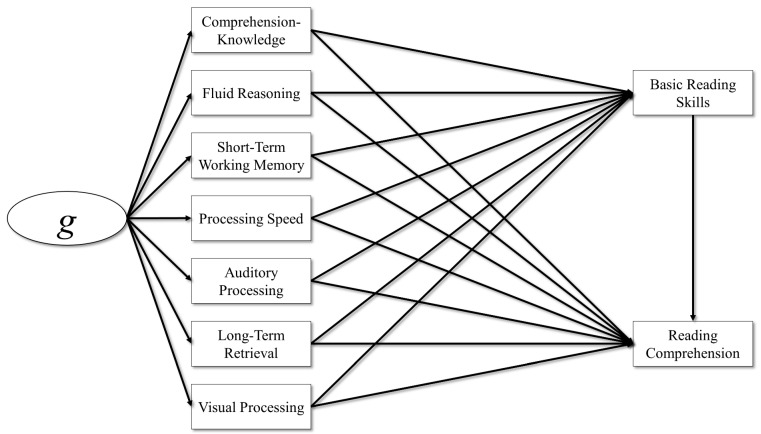
Broad CHC abilities and general intel predicting reading.

Third, a model similar to the previous model was estimated, but all of the broad CHC abilities loaded on a latent *g* factor. Here, the common variance among the broad CHC abilities is partialed out, and the independent effects of *g* and broad CHC abilities on BRS and RC can be estimated. In this model, there is no direct effect of *g* on BRS and RC. Previous research with the WJ III and WJ IV suggests that the direct effects of *g* on reading skills tend to be negative, and the effects of *g* on reading skills are indirect (Floyd et al. 2012; Niileksela et al. 2016). These negative effects between *g* and the academic skills were found when models included both direct paths from the broad abilities and *g* to the academic skill simultaneously. However, other researchers have found large and positive direct effects of *g* on reading skills (e.g., Beaujean et al. 2014; Caemmerer et al. 2018), suggesting this finding may be specific to the test battery used. The model is depicted in Figure 3.

All models were estimated using Mplus 7.4 (Muthén and Muthén 1998–2012). Maximum likelihood estimation (MLE) was used for all models to account for missing data under the assumption that data were missing at random (i.e., scores were not missing due to the individual’s level of ability on the variable with a missing score; Enders 2022). 

## 7. Results

### Descriptive Statistics

The means, standard deviations, and normality statistics for all measures from the WJ III and WJ IV used in this study for each of the ability groups are included in Table 1 and Table 2, respectively. All scores are age-based standard scores, with means of 100 and standard deviations of 15. The means for the different ability groups selected based on the altGIA were in ranges that would be expected based on the selection procedure. Most means for the Low group were in the 80s, means for the Average group were around 100, and means for the High group were around the 110s. Standard deviations tended to be between 10 and 15 for most measures, though the standard deviations for the GIA tended to be smaller. This was not unexpected given that the groups were selected on a similar measure. All test scores across batteries and ability groups were essentially normally distributed, with skewness values < |2| and kurtosis values < |7|, suggesting the use of MLE was appropriate (Curran et al. 1996).

## 8. Model Tests

### 8.1. General Intelligence Predicting Reading

The first model included the GIA as a predictor of BRS and RC and BRS as a predictor of RC. When equality constraints were added to the paths, there was a statistically significant degradation in model fit for the WJ III Elementary sample, χ^2^ (6) = 26.23, *p* < .001, WJ III Secondary sample, χ^2^ (6) = 50.64, *p* < .001, WJ IV Elementary sample, χ^2^ (6) = 28.88, *p* < .001, and WJ IV Secondary sample, χ^2^ (6) = 12.89, *p* = .045, suggesting statistically significant differences in the size of the path coefficients across the different ability groups across both the elementary and secondary samples on the WJ III and WJ IV.

Table 3 includes the unstandardized and standardized values for all paths in the model, as well as the *R*^2^ for BRS and RC in each ability group for the WJ III and WJ IV Elementary and Secondary samples. Pairwise comparisons across the Low, Average, and High groups on all paths were examined to determine where there were differences in path coefficients. For the WJ III Elementary sample, the primary difference across groups was the path from the GIA to BRS, where the value for the Low group was larger than the Average and High groups, and the value for the Average group was larger than the High group. In addition, the *R*^2^ for RC was larger for the Low group compared to the Average and High groups. 

For the WJ III Secondary sample, there were more differences across ability groups. The paths from GIA to RC and from GIA to BRS were larger for the Low group compared to the Average and High groups. The *R*^2^ for BRS in the Low group was larger than the Average group, and the *R*^2^ for RC in the Low group was larger than the Average or High groups. 

For the WJ IV Elementary sample, the path from BRS to RC was larger for the Low and Average groups compared to the High group, the path from GIA to RC was larger for the High group compared to the Low and Average groups, and the path from GIA to BRS was larger for the Average group compared to the High group. The *R*^2^ for RC in the Low group was larger than the Average or High groups. Finally, for the WJ IV Secondary sample, the path from BRS to RC was larger for the High group compared to the Average group. The path from GIA to RC was larger for the Low and Average groups compared to the High group. The *R*^2^ for RC and BRS was larger for the Low group compared to the Average group. 

### 8.2. Broad CHC Abilities Predicting Reading 

The second model included broad CHC abilities as predictors of BRS and RC, and BRS as a predictor of RC. When cross-group equality constraints were added to the paths from cognitive abilities to reading, there was not a statistically significant degradation in model fit for the WJ III Elementary sample, χ^2^ (30) = 30.00, *p* < .466, or the WJ IV Secondary sample, χ^2^ (30) = 42.86, *p* = .060. There was a statistically significant degradation in model fit for the WJ III Secondary sample, χ^2^ (30) = 69.38, *p* < .001, and the WJ IV Elementary sample, χ^2^ (30) = 72.24, *p* < .001, suggesting differences in the size of the path coefficients across the different ability groups for those samples.

Table 4 shows the results for the WJ III samples, and Table 5 shows the results for the WJ IV samples. Not surprisingly, because the χ^2^ was not statistically significant, there were few differences between groups. However, the *R*^2^ was larger for the Low group compared to the Average and High groups for both BRS and RC. In the WJ III Secondary sample, the path from G*s* to RC was larger for the Low group compared to the Average and High group, the path from G*lr* to BRS was larger for the Average group compared to the High group, and the path from G*f* to BRS was larger for the High group compared to the Average and Low groups. For BRS, the *R*^2^ values for the Low group and the High group were both larger than the Average group, and for RC, the *R*^2^ for the Low group was larger than the Average and High groups. 

The WJ IV Elementary sample had several paths that were different across ability groups. The path from BRS to RC was larger for the Low and Average groups compared to the High group. The path from G*f* to RC was larger for the Low group compared to the Average and High groups. The path from G*s* was larger for the High group compared to the Low and Average groups. The path from G*f* to BRS was larger for the Low group compared to the Average and High groups. The *R*^2^ for BRS was similar across all groups, but the *R*^2^ was larger for the Low group compared to the Average and High groups. 

Results from the WJ IV Secondary sample had few differences in the size of path coefficients across ability groups. The path from G*c* to BRS was larger for the Low group compared to the Average group, the path from G*c* to BRS was larger for the High group compared to the Average group, the path from G*a* to BRS was larger for the Low group compared to the High group, the path from G*f* to BRS was larger for the Low group compared to the Average group, and the path from G*sm* to BRS was larger for the Average group compared to the Low group. The *R*^2^ for BRS was larger for the Low group compared to the Average and High groups, and the *R*^2^ for RC was larger for the Low group compared to the Average group. 

### 8.3. Separating Effects of g and Broad Abilities Predicting Reading 

Finally, the last model was the same as the previous model, except all broad CHC abilities loaded on a single *g* factor to separate variance that can be accounted for by *g* and the broad CHC abilities in reading. In this model, all the broad CHC abilities loaded on the *g* factor, and then the broad CHC abilities predicted both BRS and RC and BRS predicted RC. When cross-group equality constraints were added to the paths from the broad CHC abilities to reading, there was not a statistically significant degradation in model fit for the WJ III Elementary sample, χ^2^ (30) = 30.81, *p* = .425, or the WJ IV Secondary sample, χ^2^ (30) = 42.86, *p* = .061. There was a statistically significant degradation in model fit for the WJ III Secondary sample, χ^2^ (30) = 69.87, *p* < .001, and the WJ IV Elementary sample, χ^2^ (30) = 72.22, *p* < .001, suggesting differences in the size of the path coefficients across the different ability groups for those samples. 

Overall, the results for path coefficients and *R*^2^ were similar to the previous model because the paths from broad CHC abilities included the indirect effects of *g* and direct effects of broad CHC abilities. All the results are in Table 6 and Table 7. To separate variance in reading accounted for by *g* and the broad CHC abilities in BRS and RC, the total indirect effect of *g* on BRS and RC was squared and then subtracted from the *R*^2^. This value represented the remaining variance accounted for by the broad CHC abilities. The square root of that value represented the total effects of the broad CHC abilities on BRS and RC. These values are included in Table 8.

For the WJ III Elementary sample, the total indirect effect of *g* on BRS was .35 for the Low group and .11 and .13 for the Average and High groups, respectively. This was larger for the Low group compared to the Average and High groups. The residualized total effects of the broad CHC abilities on BRS were .34, .31, and .29 for the Low, Average, and High groups, respectively. Importantly, the effects of *g* on BRS for the Low group was higher than the other groups, but the total effect of broad CHC abilities after removing *g* was similar across groups. The *R*^2^ differs across groups, so the relative proportion of variance accounted for in BRS for *g* and the broad CHC abilities was calculated. Here, the relative variance accounted for by *g* and the broad CHC abilities were similar for the Low group, but the relative variance accounted for by the broad CHC abilities on BRS in the Average and High groups was much different, with *g* accounting for much less variance than the broad CHC abilities. This same pattern was present for RC, and, in general, this pattern was apparent through all samples. In other words, the variance accounted for in BRS and RC by *g* was consistently larger in the Low group than the Average and High groups, and the variance accounted for in BRS and RC by the broad CHC abilities was consistently larger in the Average and High groups compared to the Low group. 

## 9. Discussion

The study of individual differences in human intelligence and its relationship with academic achievement remains an important area of inquiry. The field has recently called for more emphasis on integrated models of intelligence and achievement (e.g., Feraco et al. 2022; Hajovsky et al. 2014; Niileksela et al. 2016). However, research to date has not considered how ability level (IQ) may moderate cognitive–achievement relations. The current study examined cognitive–achievement relations using both global IQ and broad CHC cognitive abilities as predictors of basic reading skills and reading comprehension across elementary and secondary students. This study examined how these relationships differ by IQ level (low, average, and high) using the WJ III and WJ IV standardization samples. 

Overall, the findings were generally consistent across WJ III and WJ IV samples and elementary and secondary school-age cohorts. General ability tended to explain more variance in basic reading skills and reading comprehension in the Low group compared to the Average and High groups. In other words, general cognitive ability (IQ) accounted for more of the achievement score variance for those groups demonstrating lower cognitive ability. These findings are consistent with SLODR, as it was hypothesized that *g* would account for more variance in achievement outcomes for those with lower cognitive ability (and less differentiated specific cognitive abilities). Although the researchers did not examine cognitive–achievement relations as moderated by IQ level, meta-analytic work suggests that general cognitive ability has the largest direct effects on achievement (Zaboski et al. 2018).

When the broad CHC abilities were used to predict reading outcomes, the broad abilities predicted basic reading skills and reading comprehension, and basic reading skills predicted reading comprehension. There were some differences among the ability groups in which broad CHC abilities predicted reading, with some of the most consistent differences across samples being the relation from G*s* to reading comprehension, G*sm* to basic reading skills, and G*f* to basic reading skills.

Some of our findings are consistent with hypotheses proposed by the theory of mutualism. Specifically, working memory and basic reading skills relations were stronger in the WJ III secondary sample compared to the WJ III elementary sample. It has been suggested that certain cognitive abilities, like working memory and reading achievement relations, become stronger as age increases (Peng and Kievit 2020). However, SLODR would predict that these working memory and basic reading skills relations would be stronger for higher ability groups, which was not the case in all instances. We did find support for SLODR with working memory and basic reading skills in the WJ IV secondary sample, where relations were stronger for the average- to high-ability groups when compared to the low group. Prior research suggests that G*s* is both directly and indirectly related to reading comprehension, although not consistently across ages, and that it may vary based on which edition of the WJ is used (e.g., Floyd et al. 2012; Niileksela et al. 2016). Similarly, extant findings suggest that G*sm* is related to basic reading skills (Caemmerer et al. 2018; Evans et al. 2002; Floyd et al. 2007; Hajovsky et al. 2014; Niileksela et al. 2016), with G*f* showing strong associations with basic reading skills in the WJ IV (Cormier et al. 2017). 

Additionally, a consistent finding in these analyses also showed that the variance explained in basic reading skills and reading comprehension was greater for the Low group in the WJ III elementary and secondary samples. In order to examine the independent effects of *g* and broad CHC abilities on reading, we residualized the broad CHC abilities by separating variance in basic reading skills and reading comprehension attributed to *g* and broad CHC abilities (see Caemmerer et al. 2018; Hajovsky and Chesnut 2022 for other examples). By partitioning out variance attributable to *g* from the broad CHC abilities, we found evidence consistent with SLODR. Specifically, when examining the relative proportion of variance explained in reading achievement outcomes (rather than the magnitude of the path coefficients), *g* explained relatively more variance than the broad CHC abilities in both basic reading skills and reading comprehension for the Low group. These findings are consistent with studies showing that *g* tends to explain the most variance in achievement outcomes in cognitive–achievement relations research (e.g., Niileksela et al. 2016; Zaboski et al. 2018). Conversely, the broad CHC abilities explained relatively more variance than *g* in both basic reading skills and reading comprehension in the Average and High groups. These findings align with both SLODR predictions of (a) *g* explaining more variance in reading outcomes for the Low group, and (b) broad CHC abilities explaining more variance in reading outcomes for the Average and High groups. These findings are consistent with theoretical postulates according to cognitive ability differentiation (Jensen 2003). In other words, because it is theoretically posited that *g* would contribute less to the CHC broad abilities when *g* (or IQ) is higher, there is more residual variance in the higher IQ groups. This phenomenon may explain why the relative effects of the CHC broad abilities on reading achievement were generally larger in the average to higher IQ groups compared to the lower IQ groups, and why the relative effects of *g* (or IQ) on reading achievement were generally larger in the lower IQ groups compared to the average or higher IQ groups. As the proportion of variance in reading explained by the CHC broad abilities was generally larger in the average to higher IQ groups, this may also be explained according to the theory of mutualism (Zhang and Peng 2023). Mutualism theory suggests that cognitive–reading achievement relations occur in students with higher abilities and thus may show stronger mutualistic effects, especially across grade levels. Our findings corroborate some of these theoretical suppositions, as some of the cognitive–reading achievement relations were stronger in the secondary grades WJ sample. This is consistent with hypotheses noted by Peng and Kievit (2020) that suggest students with higher abilities may generate more mutualism among skills. However, we did not examine the potential for bidirectional relations in this study, which may shed light on mutualistic effects between cognitive abilities and academic achievement across ability levels. As an example, Zhang and Peng (2023) have shown evidence of mutualistic effects between verbal working memory and reading in high-math students for children in elementary school. Mutualistic effects may be most clearly seen in longitudinal studies, where relationships between growth in cognitive abilities and academic achievement can be specifically modeled and evaluated. A longitudinal study that examines the mutualistic effects of a wide range of cognitive abilities and academic achievement across time would help clarify these relationships. 

Although the findings are preliminary, the results from this study suggest that a differential interpretation of intelligence tests contingent on general ability of the tested individual (i.e., examinee) may be warranted. Identification of certain exceptionalities, such as SLD, intellectual disability, or gifted and talented considerations, may be impacted by IQ level and must be considered by researchers and practitioners. Where an individual falls on the normative IQ distribution impacts the degree to which outcomes can be explained or the strength of correlations between intelligence and achievement or progress monitoring performance over time. In other words, the relationship between two or more variables is stronger or weaker depending on where an individual falls on the IQ distribution. Examining these correlational patterns of strengths and weaknesses while considering the level of general ability may impact decisions regarding special education or disability service eligibility. 

## 10. Implications of the Findings 

The implications for students who are referred for psychoeducational evaluations for special education services under the Individuals with Education Disabilities Improvement Act (IDEIA 2004) or disability resources in post-secondary education are important. Qualified evaluators, such as psychologists, medical providers, or trained diagnostic educators, provide comprehensive sources of evaluation documentation that often include cognitive and academic assessment data. These assessment data are then used to determine special education eligibility into one of the thirteen categories of support in U.S. schools (IDEIA 2004). One of these categories is SLD, and for many states or evaluators who operate on the SLD discrepancy model or pattern of strength and weaknesses (PSW), a distinction of SLD is given to students who have an unexpected discrepancy between cognitive ability and academic performance (e.g., Maki et al. 2015). The results of this study suggest that a child’s IQ ability level has a significant impact on this relationship. This calls into question our identification methods: are we measuring a disorder, or a difference in how a child uses specific cognitive abilities? 

Given the findings of this study, practitioners need to consider IQ level in relation to student age and grade level, specifically when examining assessment data for elementary versus secondary students. This is essential when examining cognitive–achievement relations research and how it relates to SLD diagnostic accuracy. More recently, there has been a shift in using the pattern of strengths and weaknesses within evaluation. However, the impact of IQ level has not been thoroughly researched, and the findings of this study suggest a pre-existing relationship between cognitive ability and academic achievement for children with lower achievement scores. The implications of this study impact the interpretation and diagnostic considerations of these cognitive and academic scores. 

## 11. Limitations 

A limitation of this research is the demographic diversity of the normative sample. The normative sample of the Woodcock–Johnson is selective to be representative of the U.S. population, but the results cannot be generalized to English language learners (ELL), immigrants, or refugees without adequate English level proficiency and U.S. cultural exposure. Given the lack of representation of diverse language and cultural backgrounds, the results may not generalize to these minoritized U.S. groups. Future research should examine heterogeneity within the groups, such as race, ethnicity, and language moderation, with SLODR. If IQ matters in terms of prediction, then it is possible that demographic status is a moderating variable. Conducting these analyses with other cognitive and achievement assessment batteries for culturally and linguistically diverse populations is essential to promote equity for underserved U.S. populations, including how these results impact the findings of this current study. 

Further, researchers have indicated a limitation with the use of SLODR as a statistical artifact due to the influence of disturbance factors (Sorjonen and Melin 2020). For example, external factors, such as linguistic differences/confusion, illness, or individual motivation that varies in magnitude among individuals, may influence test scores and thus impact the validity of SLODR findings. In this manner, construct irrelevant variance (i.e., systematic error) related to studies of SLODR may introduce internal validity threats that are not easily controlled and thus impact the validity of inferences drawn from study results. Future studies should seek to control these possible confounding factors to more accurately assess SLODR. An additional validity concern is related to whether the constructs are being measured the same way across the different ability groups. We used observed variables in this study; therefore, we could not test the extent to which the constructs demonstrate measurement invariance across ability groups prior to testing the strength of the predictive paths. Future studies may overcome this concern by utilizing latent variable models. 

Another area for future studies is looking at cognitive–achievement relationships as they vary by achievement level using quantile regression. The model used in this study might appear more reliable for children with lower cognitive ability. Thus, future researchers should include the effects of reading, writing, or mathematical computation skills at different levels to better understand the multifaceted decisions for distinguishing between disabilities and the surrounding considerations. Finally, the integrated model used here assumed that the effects of cognitive abilities on reading comprehension are partially mediated through basic reading skills. In cross-sectional data, this does not account for the passage of time and assumes that mediation effects occur instantaneously (Cole and Maxwell 2003; Preacher 2015). Future studies can overcome this limitation by addressing these integrated models of cognitive–achievement relations through the use of longitudinal data.

## Figures and Tables

**Table 1:** Descriptive statistics for the WJ III.

	Low				Average				High			
	*N*	*M* (*SD*)	Skew	Kurt	*N*	*M* (*SD*)	Skew	Kurt	*N*	*M* (*SD*)	Skew	Kurt
**WJ III Elementary**												
G*c*	302	83.03 (11.70)	−0.64	0.48	658	101.07 (9.03)	0.07	−0.04	362	114.07 (8.57)	−0.11	0.29
G*f*	369	83.67 (13.21)	−0.64	0.18	832	100.11 (10.62)	−0.35	0.67	454	114.30 (10.84)	0.24	0.33
G*sm*	400	85.74 (13.49)	−0.13	0.87	883	100.75 (12.61)	0.01	0.28	463	111.63 (13.67)	0.50	1.33
G*s*	343	90.62 (13.87)	−0.28	0.46	701	100.98 (12.30)	−0.14	0.10	364	107.30 (14.01)	0.21	0.12
G*a*	303	89.86 (13.98)	−0.27	0.18	614	101.57 (13.07)	−0.01	0.18	337	111.96 (13.27)	0.08	0.06
G*lr*	306	87.67 (10.61)	−0.28	0.47	659	101.30 (10.29)	0.30	0.63	353	112.93 (10.70)	0.33	0.10
G*v*	277	93.28 (14.18)	−0.17	−0.34	656	99.63 (13.74)	−0.38	0.71	378	107.27 (14.13)	−0.08	1.03
BRS	409	88.11 (14.18)	−0.28	0.45	956	101.70 (11.45)	0.04	−0.08	460	112.83 (10.28)	−0.22	−0.07
RC	334	85.54 (14.26)	−0.69	1.37	795	101.82 (11.18)	−0.22	0.59	405	114.40 (11.19)	0.12	1.05
GIA	249	81.57 (8.71)	−0.80	0.99	614	99.38 (6.98)	0.07	−0.14	363	117.20 (9.53)	0.87	1.51
**WJ III Secondary**												
G*c*	326	83.26 (11.24)	−0.65	0.88	686	100.98 (9.13)	−0.40	2.14	391	115.65 (9.67)	0.48	0.42
G*f*	331	83.15 (11.79)	−0.33	0.27	704	100.24 (10.27)	−0.17	0.27	386	115.28 (9.02)	0.01	−0.05
G*sm*	426	87.62 (13.03)	−0.15	0.12	866	101.53 (11.52)	−0.02	0.37	470	112.64 (12.03)	0.11	0.15
G*s*	419	90.64 (14.92)	−0.12	0.87	849	100.12 (13.68)	−0.08	0.30	467	108.05 (14.41)	0.14	−0.05
G*a*	271	86.48 (12.37)	0.04	0.38	505	99.47 (12.20)	0.17	0.55	285	109.27 (14.21)	0.55	0.43
G*lr*	301	85.57 (11.96)	−0.17	0.23	625	98.91 (11.01)	0.47	1.31	347	112.98 (11.79)	0.62	0.30
G*v*	310	90.10 (13.00)	−0.15	−0.22	623	100.01 (12.19)	−0.21	0.40	358	109.39 (13.45)	−0.02	−0.02
BRS	457	86.57 (14.49)	−0.40	0.67	1001	101.53 (11.55)	−0.18	0.28	527	112.02 (11.92)	0.31	0.05
RC	312	84.24 (14.26)	−0.70	1.05	776	100.93 (10.78)	0.03	0.97	403	113.59 (11.74)	0.48	1.22
GIA	292	82.70 (9.52)	−1.13	1.50	604	101.79 (6.94)	0.07	−0.51	341	120.82 (9.08)	0.80	0.60

Note. G*c* = Comprehension–Knowledge, G*f* = Fluid Reasoning, G*sm* = Short-Term Memory, G*s* = Processing Speed, G*a* = Auditory Processing, G*lr* = Long-Term Retrieval, G*v* = Visual Processing, BRS = Basic Reading Skills, RC = Reading Comprehension, GIA = General Intellectual Ability.

**Table 2:** Descriptive statistics for the WJ IV.

	Low				Average				High			
	*N*	*M* (*SD*)	Skew	Kurt	*N*	*M* (*SD*)	Skew	Kurt	*N*	*M* (*SD*)	Skew	Kurt
**WJ IV Elementary**												
G*c*	400	86.27 (13.22)	0.07	−0.26	801	100.14 (12.09)	−0.12	0.69	412	111.83 (12.38)	0.11	−0.03
G*f*	401	83.95 (11.56)	−0.40	0.70	801	100.39 (9.93)	0.07	−0.02	412	115.16 (11.57)	0.25	0.10
G*wm*	400	86.03 (12.50)	−0.38	0.79	801	100.62 (11.03)	−0.09	0.20	412	114.27 (11.69)	0.00	0.78
G*s*	399	89.15 (14.33)	0.16	0.69	799	100.53 (13.05)	−0.01	0.42	412	110.04 (13.34)	0.26	0.36
G*a*	401	84.43 (12.26)	−0.07	0.88	801	99.85 (11.94)	0.13	0.17	412	114.03 (11.29)	0.07	0.19
G*lr*	401	86.13 (12.67)	−0.13	0.04	801	100.69 (12.43)	−0.21	0.20	412	112.55 (12.36)	0.13	0.20
G*v*	401	87.91 (14.01)	−0.30	0.39	801	101.13 (12.87)	0.11	0.69	412	111.95 (13.97)	0.23	0.20
BRS	401	85.93 (13.24)	−0.41	0.20	801	100.74 (11.70)	0.05	0.36	412	112.51 (11.99)	0.23	0.29
RC	396	85.96 (14.43)	−0.79	1.39	800	101.26 (11.79)	−0.07	0.54	412	113.71 (12.55)	0.33	0.22
GIA	401	80.14 (9.57)	−1.06	1.49	801	100.39 (6.45)	0.13	−0.34	412	118.37 (7.62)	1.23	2.48
**WJ IV Secondary**												
G*c*	466	86.43 (12.77)	−0.01	0.48	997	99.72 (11.66)	0.11	0.19	492	115.14 (12.71)	0.42	0.18
G*f*	466	82.82 (11.68)	−0.33	0.21	997	99.81 (11.32)	0.00	0.28	492	114.04 (10.91)	0.08	−0.16
G*wm*	466	85.97 (11.31)	−0.09	0.24	997	100.54 (11.99)	0.04	0.00	492	115.16 (11.73)	0.22	−0.06
G*s*	466	87.59 (14.95)	−0.40	0.66	997	99.60 (12.81)	−0.10	0.13	492	110.45 (12.69)	−0.10	−0.22
G*a*	466	84.55 (11.85)	−0.09	0.18	997	100.47 (11.46)	0.18	−0.26	492	114.14 (12.62)	0.20	−0.01
G*lr*	466	87.11 (13.97)	−0.12	0.16	997	100.35 (12.34)	0.17	0.06	492	112.35 (13.18)	0.15	−0.13
G*v*	466	88.71 (14.34)	−0.05	0.51	997	101.07 (13.03)	0.17	0.34	492	111.13 (14.02)	0.31	0.07
BRS	466	86.04 (13.62)	−0.47	1.01	997	100.22 (12.29)	0.24	0.82	492	113.71 (13.69)	0.26	0.29
RC	466	83.87 (14.84)	−0.58	0.81	997	100.09 (12.49)	0.04	0.32	492	112.32 (13.48)	−0.05	0.35
GIA	466	79.28 (9.57)	−1.24	2.22	997	99.52 (6.90)	0.02	−0.54	492	118.21 (7.66)	0.67	0.27

Note. G*c* = Comprehension–Knowledge, G*f* = Fluid Reasoning, G*wm* = Short-Term Working Memory, G*s* = Processing Speed, G*a* = Auditory Processing, G*lr* = Long-Term Retrieval, G*v* = Visual Processing, BRS = Basic Reading Skills, RC = Reading Comprehension, GIA = General Intellectual Ability.

**Table 3:** GIA predicting reading.

	Low		Average		High		Pairwise Comparisons
	*b* (*SE*)	*β* (*SE*)	*b* (*SE*)	*β* (*SE*)	*b* (*SE*)	*β* (*SE*)	
WJ III Elementary							
BRS→RC	.57 (.05)	.56 (.04)	.46 (.03)	.48 (.03)	.48 (.05)	.44 (.04)	L = A, L = H, A = H
GIA→RC	.36 (.09)	.22 (.05)	.23 (.06)	.15 (.04)	.29 (.06)	.25 (.05)	L = A, L = H, A = H
GIA→BRS	.70 (.09)	.43 (.05)	.46 (.07)	.28 (.04)	.25 (.06)	.23 (.06)	L > A, L > H, A > H
BRS *R*^2^	.19 (.05)		.08 (.02)		.06 (.03)		L = A, L = H, A = H
RC *R*^2^	.47 (.04)		.29 (.03)		.30 (.04)		L > A, L > H, A = H
WJ III Secondary							
BRS→RC	.30 (.05)	.30 (.05)	.26 (.03)	.27 (.04)	.32 (.05)	.32 (.05)	L = A, L = H, A = H
GIA→RC	.77 (.08)	.52 (.05)	.35 (.07)	.22 (.04)	.22 (.08)	.17 (.06)	L > A, L > H, A = H
GIA→BRS	.79 (.07)	.52 (.04)	.48 (.06)	.29 (.04)	.52 (.06)	.40 (.05)	L > A, L > H, A = H
BRS *R*^2^	.27 (.04)		.08 (.02)		.16 (.04)		L > A, L = H, A = H
RC *R*^2^	.53 (.04)		.16 (.03)		.18 (.04)		L > A, L > H, A = H
WJ IV Elementary							
BRS→RC	.67 (.04)	.61 (.03)	.59 (.03)	.58 (.03)	.41 (.05)	.39 (.04)	L = A, L > H, A > H
GIA→RC	.40 (.06)	.26 (.04)	.31 (.05)	.17 (.03)	.56 (.07)	.34 (.04)	L = A, L = H, A < H
GIA→BRS	.78 (.06)	.57 (.03)	.89 (.06)	.49 (.03)	.69 (.07)	.44 (.04)	L = A, L = H, A > H
BRS *R*^2^	.32 (.04)		.24 (.03)		.19 (.04)		L = A, L = H, A = H
RC *R*^2^	.62 (.03)		.47 (.03)		.38 (.04)		L > A, L > H, A = H
WJ IV Secondary							
BRS→RC	.46 (.04)	.43 (.04)	.40 (.03)	.39 (.03)	.49 (.04)	.50 (.04)	L = A, L = H, A < H
GIA→RC	.58 (.06)	.38 (.04)	.54 (.05)	.30 (.03)	.36 (.07)	.20 (.04)	L = A, L > H, A > H
GIA→BRS	.77 (.06)	.54 (.03)	.66 (.05)	.37 (.03)	.71 (.07)	.40 (.04)	L = A, L = H, A = H
BRS *R*^2^	.29 (.04)		.14 (.02)		.16 (.03)		L > A, L = H, A = H
RC *R*^2^	.50 (.03)		.33 (.02)		.37 (.04)		L > A, L = H, A = H

Note. All values are statistically significant (*p* < .05). BRS = Basic Reading Skills, RC = Reading Comprehension, GIA = General Intellectual Ability, L = Low, A = Average, H = High.

**Table 4:** WJ III broad abilities predicting reading.

	Low		Average		High		Pairwise Comparisons
	*b* (*SE*)	*β* (*SE*)	*b* (*SE*)	*β* (*SE*)	*b* (*SE*)	*β* (*SE*)	
**WJ III Elementary**							
BRS→RC	**.50 (.05)**	**.49 (.04)**	**.44 (.03)**	**.45 (.03)**	**.45 (.05)**	**.41 (.04)**	L = A, L = H, A = H
G*c*→RC	**.36 (.06)**	**.29 (.05)**	**.34 (.04)**	**.28 (.03)**	**.38 (.06)**	**.29 (.05)**	L = A, L = H, A = H
G*lr*→RC	*.10 (.07)*	*.08 (.05)*	**.11 (.04)**	**.10 (.04)**	*.09 (.05)*	*.09 (.05)*	L = A, L = H, A = H
G*v*→RC	*−.07 (.05)*	*−.06 (.05)*	*−.03 (.03)*	*−.03 (.04)*	*.03 (.04)*	*.04 (.05)*	L = A, L = H, A = H
G*a*→RC	*.00 (.06)*	*.00 (.06)*	*.02 (.04)*	*.03 (.05)*	*.04 (.05)*	*.04 (.06)*	L = A, L = H, A = H
G*f*→RC	*.03 (.05)*	*.02 (.04)*	**.07 (.03)**	**.07 (.03)**	*.03 (.05)*	*.03 (.04)*	L = A, L = H, A = H
G*s*→RC	**.11 (.05)**	**.11 (.05)**	*−.01 (.03)*	*−.01 (.04)*	*.04 (.04)*	*.05 (.05)*	L > A, L = H, A = H
G*sm*→RC	*.04 (.05)*	*.03 (.04)*	*−.02 (.03)*	*−.02 (.03)*	*.02 (.04)*	*.02 (.05)*	L = A, L = H, A = H
G*c*→BRS	**.32 (.07)**	**.26 (.06)**	**.28 (.05)**	**.22 (.04)**	**.20 (.06)**	**.17 (.05)**	L = A, L = H, A = H
G*lr*→BRS	**.21 (.09)**	**.16 (.07)**	*.06 (.05)*	*.06 (.04)*	*.04 (.06)*	*.05 (.06)*	L = A, L = H, A = H
G*v*→RS	*−.04 (.06)*	*−.04 (.06)*	*.02 (.04)*	*.03 (.05)*	*−.01 (.04)*	*−.01 (.06)*	L = A, L = H, A = H
G*a*→BRS	*.03 (.07)*	*.03 (.07)*	*.05 (.04)*	*.06 (.05)*	*.02 (.05)*	*.03 (.07)*	L = A, L = H, A = H
G*f*→BRS	*.00 (.06)*	*.00 (.06)*	*−.05 (.04)*	*−.05 (.04)*	*.00 (.05)*	*.00 (.05)*	L = A, L = H, A = H
G*s*→BRS	*.09 (.06)*	*.09 (.06)*	**.16 (.03)**	**.17 (.04)**	*.05 (.04)*	*.07 (.06)*	L = A, L = H, A > H
G*sm*→BRS	**.22 (.05)**	**.21 (.05)**	**.11 (.03)**	**.13 (.04)**	**.17 (.04)**	**.23 (.05)**	L = A, L = H, A = H
BRS *R*^2^	.24 (.04)		.11 (.02)		.10 (.03)		L > A, L > H, A = H
RC *R*^2^	.54 (.04)		.36 (.03)		.34 (.04)		L > A, L > H, A = H
**WJ III Secondary**							
BRS→RC	**.27 (.04)**	**.28 (.05)**	**.17 (.03)**	**.18 (.03)**	**.15 (.05)**	**.16 (.05)**	L = A, L = H, A = H
G*c*→RC	**.55 (.06)**	**.44 (.05)**	**.53 (.04)**	**.45 (.03)**	**.55 (.06)**	**.45 (.05)**	L = A, L = H, A = H
G*lr*→RC	*.04 (.06)*	*.03 (.05)*	**.10 (.04)**	**.10 (.04)**	*.03 (.05)*	*.03 (.05)*	L = A, L = H, A = H
G*v*→RC	*.00 (.05)*	*.00 (.05)*	*−.04 (.03)*	*−.05 (.04)*	*.06 (.04)*	*.06 (.05)*	L = A, L = H, A = H
G*a*→RC	*.06 (.07)*	*.06 (.06)*	*.00 (.04)*	*.00 (.05)*	*−.01 (.05)*	*−.01 (.06)*	L = A, L = H, A = H
G*f*→RC	**.15 (.06)**	**.13 (.05)**	**.12 (.04)**	**.12 (.04)**	*.05 (.06)*	*.04 (.05)*	L = A, L = H, A = H
G*s*→RC	**.10 (.05)**	**.11 (.05)**	*−.02 (.03)*	*−.03 (.04)*	*−.03 (.04)*	*−.04 (.05)*	L > A, L > H, A = H
G*sm*→RC	**.13 (.05)**	**.12 (.05)**	*.02 (.03)*	*.03 (.03)*	*.06 (.05)*	*.06 (.05)*	L = A, L = H, A = H
G*c*→BRS	**.36 (.06)**	**.28 (.05)**	**.41 (.04)**	**.32 (.03)**	**.50 (.05)**	**.41 (.04)**	L = A, L = H, A = H
G*lr*→BRS	*−.02 (.07)*	*−.02 (.06)*	*.04 (.04)*	*.04 (.04)*	*−.09 (.05)*	*−.09 (.05)*	L = A, L = H, A > H
G*v*→RS	*.04 (.06)*	*.03 (.05)*	*−.06 (.04)*	*−.06 (.04)*	*.05 (.04)*	*.05 (.05)*	L = A, L = H, A = H
G*a*→BRS	*.08 (.07)*	*.07 (.06)*	**.09 (.04)**	**.09 (.05)**	*.05 (.04)*	*.06 (.05)*	L = A, L = H, A = H
G*f*→BRS	*−.05 (.06)*	*−.04 (.05)*	*−.05 (.04)*	*−.04 (.04)*	**.14 (.06)**	**.11 (.05)**	L = A, L < H, A < H
G*s*→BRS	**.11 (.05)**	**.11 (.05)**	*.05 (.03)*	*.06 (.03)*	**.08 (.03)**	**.10 (.04)**	L = A, L = H, A = H
G*sm*→BRS	**.41 (.05)**	**.37 (.04)**	**.21 (.03)**	**.21 (.03)**	**.18 (.04)**	**.19 (.04)**	L > A, L > H, A = H
BRS *R*^2^	.31 (.04)		.16 (.03)		.29 (.04)		L > A, L = H, A < H
RC *R*^2^	.59 (.04)		.34 (.03)		.34 (.04)		L > A, L > H, A = H

Note. Values in bold are statistically significant; values in italics are not statistically significant (*p* < .05). G*c* = Comprehension–Knowledge, G*f* = Fluid Reasoning, G*sm* = Short-Term Memory, G*s* = Processing Speed, G*a* = Auditory Processing, G*lr* = Long-Term Retrieval, G*v* = Visual Processing, BRS = Basic Reading Skills, RC = Reading Comprehension, L = Low, A = Average, H = High.

**Table 5:** WJ IV broad abilities predicting reading.

	Low		Average		High		Pairwise Comparisons
	*b* (*SE*)	*β* (*SE*)	*b* (*SE*)	*β* (*SE*)	*b* (*SE*)	*β* (*SE*)	
**WJ IV Elementary**							
BRS→RC	**.65 (.04)**	**.59 (.03)**	**.59 (.03)**	**.58 (.03)**	**.44 (.05)**	**.42 (.04)**	L = A, L > H, A > H
G*c*→RC	**.09 (.04)**	**.08 (.03)**	**.10 (.03)**	**.10 (.03)**	**.09 (.04)**	**.08 (.04)**	L = A, L = H, A = H
G*lr*→RC	*.04 (.04)*	*.04 (.03)*	**−.06 (.03)**	**−.06 (.03)**	*.01 (.04)*	*.01 (.04)*	L > A, L = H, A = H
G*v*→RC	*.03 (.04)*	*.03 (.03)*	*.04 (.03)*	*.05 (.03)*	*.03 (.04)*	*.03 (.04)*	L = A, L = H, A = H
G*a*→RC	*−.07 (.04)*	*−.06 (.03)*	*.00 (.03)*	*.00 (.03)*	*.01 (.05)*	*.01 (.04)*	L = A, L = H, A = H
G*f*→RC	**.44 (.04)**	**.35 (.03)**	**.20 (.03)**	**.17 (.03)**	**.28 (.05)**	**.26 (.04)**	L > A, L > H, A = H
G*s*→RC	*−.03 (.03)*	*−.03 (.03)*	*−.05 (.02)*	*−.05 (.03)*	**.08 (.04)**	**.08 (.04)**	L = A, L < H, A < H
G*sm*→RC	*−.06 (.04)*	*−.05 (.03)*	*−.01 (.03)*	*−.01 (.03)*	*−.01 (.04)*	*−.01 (.04)*	L = A, L = H, A = H
G*c*→BRS	**.21 (.05)**	**.21 (.05)**	**.21 (.03)**	**.22 (.03)**	**.19 (.04)**	**.19 (.04)**	L = A, L = H, A = H
G*lr*→BRS	*−.08 (.05)*	*−.08 (.05)*	**−.13 (.03)**	**−.14 (.03)**	**−.11 (.04)**	**−.11 (.05)**	L = A, L = H, A = H
G*v*→RS	*.02 (.05)*	*.03 (.05)*	**.06 (.03)**	**.07 (.03)**	*.06 (.04)*	*.07 (.05)*	L = A, L = H, A = H
G*a*→BRS	**.15 (.05)**	**.14 (.05)**	**.14 (.03)**	**.14 (.03)**	**.17 (.05)**	**.16 (.05)**	L = A, L = H, A = H
G*f*→BRS	**.47 (.05)**	**.41 (.04)**	**.31 (.04)**	**.27 (.03)**	**.30 (.05)**	**.29 (.04)**	L > A, L > H, A = H
G*s*→BRS	*.00 (.04)*	*.00 (.04)*	*.02 (.03)*	*.03 (.03)*	*.01 (.04)*	*.01 (.05)*	L = A, L = H, A = H
G*sm*→BRS	*.02 (.05)*	*.02 (.05)*	**.16 (.03)**	**.15 (.03)**	*.09 (.05)*	*.09 (.05)*	L < A, L = H, A = H
BRS *R*^2^	.31 (.04)		.22 (.03)		.22 (.04)		L = A, L = H, A = H
RC *R*^2^	.68 (.03)		.49 (.03)		.37 (.04)		L > A, L > H, A = H
**WJ IV Secondary**							
BRS→RC	**.54 (.04)**	**.49 (.04)**	**.44 (.03)**	**.44 (.03)**	**.52 (.04)**	**.53 (.03)**	L = A, L = H, A = H
G*c*→RC	**.17 (.04)**	**.15 (.04)**	**.15 (.03)**	**.14 (.03)**	**.13 (.04)**	**.13 (.04)**	L = A, L = H, A = H
G*lr*→RC	**.12 (.04)**	**.11 (.04)**	**.06 (.03)**	**.06 (.03)**	*.07 (.04)*	*.07 (.04)*	L = A, L = H, A = H
G*v*→RC	*.03 (.04)*	*.03 (.03)*	*.05 (.03)*	*.05 (.03)*	*.01 (.04)*	*.01 (.04)*	L = A, L = H, A = H
G*a*→RC	*−.06 (.05)*	*−.05 (.04)*	*−.05 (.03)*	*−.05 (.03)*	**−.09 (.04)**	**−.09 (.04)**	L = A, L = H, A = H
G*f*→RC	**.33 (.05)**	**.26 (.04)**	**.31 (.03)**	**.28 (.03)**	**.31 (.04)**	**.25 (.04)**	L = A, L = H, A = H
G*s*→RC	*.03 (.03)*	*.03 (.03)*	*−.01 (.03)*	*−.01 (.03)*	*−.04 (.04)*	*−.04 (.03)*	L = A, L = H, A = H
G*sm*→RC	*−.04 (.05)*	*−.03 (.04)*	*−.02 (.03)*	*−.02 (.03)*	*−.04 (.04)*	*−.04 (.04)*	L = A, L = H, A = H
G*c*→BRS	**.30 (.04)**	**.28 (.04)**	**.17 (.03)**	**.16 (.03)**	**.20 (.05)**	**.18 (.04)**	L > A, L = H, A = H
G*lr*→BRS	**−.17 (.04)**	**−.18 (.04)**	**−.11 (.03)**	**−.11 (.03)**	**−.17 (.05)**	**−.16 (.04)**	L = A, L = H, A = H
G*v*→RS	*.03 (.04)*	*.03 (.04)*	*.02 (.03)*	*.02 (.03)*	**.13 (.04)**	**.14 (.04)**	L = A, L = H, A < H
G*a*→BRS	**.27 (.05)**	**.23 (.04)**	**.17 (.03)**	**.16 (.03)**	**.13 (.05)**	**.12 (.04)**	L = A, L > H, A = H
G*f*→BRS	**.34 (.05)**	**.29 (.04)**	**.20 (.03)**	**.19 (.03)**	**.24 (.05)**	**.19 (.04)**	L > A, L = H, A = H
G*s*→BRS	**.09 (.04)**	**.10 (.04)**	*.05 (.03)*	*.05 (.03)*	*.01 (.05)*	*.01 (.04)*	L = A, L = H, A = H
G*sm*→BRS	*.02 (.05)*	*.01 (.04)*	**.14 (.03)**	**.14 (.03)**	**.14 (.05)**	**.12 (.04)**	L < A, L = H, A = H
BRS *R*^2^	.32 (.04)		.14 (.02)		.16 (.03)		L > A, L > H, A = H
RC *R*^2^	.51 (.03)		.37 (.02)		.44 (.03)		L > A, L = H, A = H

Note. Values in bold are statistically significant; values in italics are not statistically significant (*p* < .05). G*c* = Comprehension–Knowledge, G*f* = Fluid Reasoning, G*sm* = Short-term Working Memory, G*s* = Processing Speed, G*a* = Processing Speed, G*lr* = Long-Term Retrieval, G*v* = Visual Processing, BRS = Basic Reading Skills, RC = Reading Comprehension, L = Low, A = Average, H = High.

**Table 6:** WJ III broad abilities and *g* predicting reading.

	Low		Average		High		Pairwise Comparisons
	*b* (*SE*)	*β* (*SE*)	*b* (*SE*)	*β* (*SE*)	*b* (*SE*)	*β* (*SE*)	
**WJ III Elementary**							
BRS→RC	**.49 (.05)**	**.49 (.04)**	**.44 (.03)**	**.45 (.03)**	**.45 (.05)**	**.41 (.04)**	L = A, L = H, A = H
G*c*→RC	**.36 (.06)**	**.30 (.05)**	**.34 (.04)**	**.28 (.03)**	**.37 (.06)**	**.29 (.04)**	L = A, L = H, A = H
G*lr*→RC	*.10 (.07)*	*.08 (.05)*	**.11 (.04)**	**.10 (.04)**	*.09 (.05)*	*.08 (.05)*	L = A, L = H, A = H
G*v*→RC	*−.06 (.05)*	*−.06 (.05)*	*−.03 (.03)*	*−.03 (.04)*	*.04 (.04)*	*.05 (.05)*	L = A, L = H, A = H
G*a*→RC	*.01 (.06)*	*.01 (.06)*	*.02 (.04)*	*.03 (.04)*	*.03 (.05)*	*.04 (.06)*	L = A, L = H, A = H
G*f*→RC	*.02 (.05)*	*.02 (.05)*	**.07 (.03)**	**.07 (.03)**	*.02 (.05)*	*.02 (.04)*	L = A, L = H, A = H
G*s*→RC	**.11 (.05)**	**.11 (.05)**	*−.01 (.03)*	*−.01 (.04)*	*.05 (.04)*	*.06 (.05)*	L > A, L = H, A = H
G*sm*→RC	*.04 (.05)*	*.03 (.04)*	*−.02 (.03)*	*−.02 (.03)*	*.02 (.04)*	*.02 (.05)*	L = A, L = H, A = H
G*c*→BRS	**.32 (.07)**	**.27 (.06)**	**.28 (.05)**	**.22 (.04)**	**.20 (.06)**	**.17 (.05)**	L = A, L = H, A = H
G*lr*→BRS	**.20 (.09)**	**.15 (.07)**	*.06 (.05)*	*.06 (.04)*	*.05 (.05)*	*.05 (.06)*	L = A, L = H, A = H
G*v*→RS	*−.03 (.06)*	*−.03 (.06)*	*.02 (.04)*	*.03 (.04)*	*−.01 (.04)*	*−.01 (.06)*	L = A, L = H, A = H
G*a*→BRS	*.02 (.06)*	*.02 (.06)*	*.06 (.04)*	*.06 (.05)*	*.01 (.05)*	*.02 (.07)*	L = A, L = H, A = H
G*f*→BRS	*−.01 (.06)*	*−.01 (.06)*	*−.05 (.04)*	*−.05 (.04)*	*.00 (.05)*	*.00 (.05)*	L = A, L = H, A = H
G*s*→BRS	*.10 (.06)*	*.09 (.05)*	**.16 (.03)**	**.17 (.04)**	*.05 (.04)*	*.07 (.05)*	L = A, L = H, A > H
G*sm*→BRS	**.22 (.05)**	**.21 (.05)**	**.11 (.03)**	**.12 (.03)**	**.18 (.04)**	**.23 (.05)**	L = A, L = H, A = H
*g*→BRS	**5.00 (.63)**	**.35 (.04)**	**3.52 (.57)**	**.11 (.02)**	**2.90 (.64)**	**.13 (.03)**	L > A, L > H, A = H
*g*→RC	**6.16 (.65)**	**.43 (.04)**	**4.86 (.59)**	**.16 (.03)**	**5.09 (.73)**	**.21 (.04)**	L > A, L > H, A = H
BRS *R*^2^	.24 (.04)		.11 (.02)		.10 (.03)		L > A, L > H, A = H
RC *R*^2^	.54 (.04)		.36 (.03)		.34 (.04)		L > A, L > H, A = H
**WJ III Secondary**							
BRS→RC	**.27 (.04)**	**.27 (.05)**	**.17 (.03)**	**.18 (.03)**	**.15 (.05)**	**.16 (.05)**	L = A, L = H, A = H
G*c*→RC	**.55 (.06)**	**.46 (.05)**	**.53 (.04)**	**.46 (.03)**	**.55 (.06)**	**.46 (.05)**	L = A, L = H, A = H
G*lr*→RC	*.04 (.06)*	*.03 (.05)*	**.10 (.04)**	**.10 (.04)**	*.03 (.05)*	*.03 (.05)*	L = A, L = H, A = H
G*v*→RC	*.01 (.05)*	*.01 (.05)*	*−.04 (.03)*	*−.04 (.04)*	*.06 (.04)*	*.06 (.05)*	L = A, L = H, A = H
G*a*→RC	*.06 (.07)*	*.06 (.06)*	*.01 (.04)*	*.01 (.04)*	*−.01 (.05)*	*−.01 (.06)*	L = A, L = H, A = H
G*f*→RC	**.15 (.05)**	**.12 (.05)**	**.12 (.04)**	**.12 (.04)**	*.05 (.06)*	*.04 (.05)*	L = A, L = H, A = H
G*s*→RC	**.10 (.04)**	**.10 (.04)**	*−.02 (.03)*	*−.03 (.03)*	*−.03 (.04)*	*−.04 (.05)*	L > A, L > H, A = H
G*sm*→RC	**.14 (.05)**	**.12 (.05)**	*.02 (.03)*	*.02 (.03)*	*.06 (.04)*	*.06 (.05)*	L = A, L = H, A = H
G*c*→BRS	**.37 (.06)**	**.30 (.05)**	**.41 (.04)**	**.32 (.03)**	**.50 (.05)**	**.42 (.04)**	L = A, L = H, A = H
G*lr*→BRS	*−.03 (.07)*	*−.02 (.05)*	*.04 (.04)*	*.04 (.04)*	*−.09 (.05)*	*−.09 (.05)*	L = A, L = H, A > H
G*v*→RS	*.04 (.06)*	*.04 (.05)*	*−.05 (.04)*	*−.06 (.04)*	*.04 (.04)*	*.05 (.05)*	L = A, L = H, A = H
G*a*→BRS	*.09 (.07)*	*.07 (.06)*	**.08 (.04)**	**.09 (.04)**	*.05 (.04)*	*.06 (.05)*	L = A, L = H, A = H
G*f*→BRS	*−.05 (.06)*	*−.04 (.05)*	*−.06 (.04)*	*−.05 (.04)*	**.14 (.06)**	**.11 (.05)**	L = A, L < H, A < H
G*s*→BRS	**.10 (.04)**	**.11 (.04)**	*.05 (.03)*	*.06 (.03)*	**.08 (.03)**	**.10 (.04)**	L = A, L = H, A > H
G*sm*→BRS	**.41 (.05)**	**.36 (.04)**	**.21 (.03)**	**.21 (.03)**	**.19 (.04)**	**.19 (.04)**	L > A, L > H, A = H
*g*→BRS	**5.21 (.60)**	**.36 (.04)**	**4.14 (.55)**	**.15 (.02)**	**5.69 (.65)**	**.28 (.04)**	
*g*→RC	**7.85 (.65)**	**.55 (.03)**	**5.66 (.56)**	**.22 (.03)**	**5.54 (.69)**	**.28 (.04)**	
BRS *R*^2^	.32 (.04)		.18 (.03)		.29 (.04)		L > A, L = H, A = H
RC *R*^2^	.61 (.04)		.35 (.03)		.33 (.04)		L > A, L > H, A = H

Note. Values in bold are statistically significant; values in italics are not statistically significant (*p* < .05). G*c* = Comprehension–Knowledge, G*f* = Fluid Reasoning, G*sm* = Short-Term Memory, G*s* = Processing Speed, G*a* = Auditory Processing, G*lr* = Long-Term Retrieval, G*v* = Visual Processing, BRS = Basic Reading Skills, RC = Reading Comprehension, L = Low, A = Average, H = High.

**Table 7:** WJ IV residualized broad abilities predicting reading.

	Low		Average		High		Pairwise Comparisons
	*b* (*SE*)	*β* (*SE*)	*b* (*SE*)	*β* (*SE*)	*b* (*SE*)	*β* (*SE*)	
**WJ IV Elementary**							
BRS→RC	**.65 (.04)**	**.59 (.03)**	**.59 (.03)**	**.58 (.03)**	**.44 (.05)**	**.42 (.04)**	L = A, L > H, A > H
G*c*→RC	**.09 (.04)**	**.08 (.03)**	**.10 (.03)**	**.10 (.03)**	**.09 (.04)**	**.08 (.04)**	L = A, L = H, A = H
G*lr*→RC	*.04 (.04)*	*.04 (.03)*	**−.06 (.03)**	**−.06 (.03)**	*.01 (.04)*	*.01 (.04)*	L > A, L = H, A = H
G*v*→RC	*.03 (.04)*	*.03 (.03)*	*.04 (.03)*	*.05 (.03)*	*.03 (.04)*	*.03 (.04)*	L = A, L = H, A = H
G*a*→RC	*−.07 (.04)*	*−.06 (.03)*	*.00 (.03)*	*.00 (.03)*	*.01 (.05)*	*.01 (.04)*	L = A, L = H, A = H
G*f*→RC	**.44 (.04)**	**.35 (.03)**	**.20 (.03)**	**.17 (.03)**	**.28 (.05)**	**.26 (.04)**	L > A, L > H, A = H
G*s*→RC	*−.03 (.03)*	*−.03 (.03)*	**−.05 (.02)**	**−.05 (.03)**	**.08 (.04)**	**.08 (.04)**	L = A, L < H, A < H
G*sm*→RC	*−.06 (.04)*	*−.05 (.03)*	*−.01 (.03)*	*−.01 (.03)*	*−.01 (.04)*	*−.01 (.04)*	L = A, L = H, A = H
G*c*→BRS	**.21 (.05)**	**.21 (.04)**	**.21 (.03)**	**.22 (.03)**	**.19 (.04)**	**.19 (.04)**	L = A, L = H, A = H
G*lr*→BRS	*−.08 (.05)*	*−.08 (.05)*	**−.13 (.03)**	**−.14 (.03)**	**−.11 (.04)**	**−.11 (.05)**	L = A, L = H, A = H
G*v*→RS	*.02 (.05)*	*.02 (.05)*	**.06 (.03)**	**.07 (.03)**	*.06 (.04)*	*.07 (.05)*	L = A, L = H, A = H
G*a*→BRS	**.15 (.05)**	**.14 (.05)**	**.14 (.03)**	**.14 (.03)**	**.17 (.05)**	**.16 (.05)**	L = A, L = H, A = H
G*f*→BRS	**.47 (.05)**	**.42 (.04)**	**.31 (.04)**	**.27 (.03)**	**.30 (.05)**	**.29 (.04)**	L > A, L > H, A = H
G*s*→BRS	*.00 (.04)*	*.00 (.04)*	*.02 (.03)*	*.03 (.03)*	*.01 (.04)*	*.01 (.05)*	L = A, L = H, A > H
G*sm*→BRS	*.02 (.05)*	*.02 (.05)*	**.16 (.03)**	**.15 (.03)**	*.09 (.05)*	*.09 (.05)*	L < A, L = H, A = H
*g*→BRS	**4.82 (.52)**	**.36 (.03)**	**4.59 (.48)**	**.21 (.02)**	**4.25 (.56)**	**.25 (.03)**	
*g*→RC	**5.83 (.56)**	**.39 (.03)**	**4.20 (.47)**	**.19 (.02)**	**4.67 (.59)**	**.26 (.03)**	
BRS *R*^2^	.31 (.04)		.22 (.03)		.21 (.04)		L > A, L > H, A = H
RC *R*^2^	.68 (.03)		.49 (.03)		.38 (.04)		L > A, L > H, A = H
**WJ IV Secondary**							
BRS→RC	**.54 (.04)**	**.50 (.04)**	**.44 (.03)**	**.44 (.03)**	**.52 (.04)**	**.53 (.03)**	L = A, L = H, A = H
G*c*→RC	**.17 (.04)**	**.15 (.04)**	**.15 (.03)**	**.14 (.03)**	**.13 (.04)**	**.12 (.04)**	L = A, L = H, A = H
G*lr*→RC	**.12 (.04)**	**.11 (.04)**	**.06 (.03)**	**.06 (.03)**	*.07 (.04)*	*.07 (.04)*	L = A, L = H, A = H
G*v*→RC	*.03 (.04)*	*.03 (.03)*	*.05 (.03)*	*.05 (.03)*	*.01 (.04)*	*.01 (.04)*	L = A, L = H, A = H
G*a*→RC	*−.06 (.05)*	*−.05 (.04)*	*−.05 (.03)*	*−.05 (.03)*	**−.09 (.04)**	**−.09 (.04)**	L = A, L = H, A = H
G*f*→RC	**.33 (.05)**	**.25 (.04)**	**.31 (.03)**	**.28 (.03)**	**.31 (.04)**	**.26 (.04)**	L = A, L = H, A = H
G*s*→RC	*.03 (.03)*	*.03 (.03)*	*−.01 (.03)*	*−.01 (.03)*	*−.04 (.04)*	*−.04 (.03)*	L = A, L = H, A = H
G*sm*→RC	*−.04 (.05)*	*−.03 (.04)*	*−.02 (.03)*	*−.02 (.03)*	*−.04 (.04)*	*−.04 (.04)*	L = A, L = H, A = H
G*c*→BRS	**.30 (.04)**	**.28 (.04)**	**.17 (.03)**	**.16 (.03)**	**.20 (.05)**	**.18 (.04)**	L > A, L = H, A = H
G*lr*→BRS	**−.17 (.04)**	**−.17 (.04)**	**−.11 (.03)**	**−.11 (.03)**	**−.17 (.05)**	**−.16 (.04)**	L = A, L = H, A = H
G*v*→RS	*.03 (.04)*	*.03 (.04)*	*.02 (.03)*	*.02 (.03)*	**.13 (.04)**	**.13 (.04)**	L = A, L = H, A < H
G*a*→BRS	**.27 (.05)**	**.23 (.04)**	**.17 (.03)**	**.16 (.03)**	**.13 (.05)**	**.12 (.04)**	L = A, L > H, A = H
G*f*→BRS	**.34 (.05)**	**.29 (.04)**	**.20 (.03)**	**.19 (.03)**	**.24 (.05)**	**.20 (.04)**	L > A, L = H, A = H
G*s*→BRS	**.09 (.04)**	**.10 (.04)**	*.05 (.03)*	*.05 (.03)*	*.01 (.05)*	*.01 (.04)*	L = A, L = H, A > H
G*sm*→BRS	*.02 (.05)*	*.01 (.04)*	**.14 (.03)**	**.14 (.03)**	**.14 (.05)**	**.12 (.04)**	L < A, L = H, A = H
*g*→BRS	**4.88 (.49)**	**.35 (.03)**	**3.55 (.41)**	**.15 (.02)**	**3.78 (.54)**	**.21 (.03)**	
*g*→RC	**5.76 (.55)**	**.39 (.03)**	**4.27 (.43)**	**.18 (.02)**	**3.99 (.53)**	**.22 (.03)**	
BRS *R*^2^	.33 (.04)		.13 (.02)		.17 (.03)		L > A, L = H, A = H
RC *R*^2^	.51 (.03)		.37 (.02)		.44 (.03)		L > A, L > H, A = H

Note. Values in bold are statistically significant; values in italics are not statistically significant (*p* < .05). G*c* = Comprehension–Knowledge, G*f* = Fluid Reasoning, G*sm* = Short-Term Working Memory, G*s* = Processing Speed, G*a* = Auditory Processing, G*lr* = Long-Term Retrieval, G*v* = Visual Processing, BRS = Basic Reading Skills, RC = Reading Comprehension, L = Low, A = Average, H = High.

**Table 8:** Effects of *g* and broad CHC abilities on reading.

	*R* ^2^	Total Indirect Effect of *g* (Effect^2^)	Total Effects of Broad CHC Abilities (Effect^2^)	Relative Variance Accounted for by *g*	Relative Variance Accounted for by Broad CHC Abilities
WJ III Elementary					
BRS					
Low	.24	.35 (.12)	.34 (.12)	.51	.49
Average	.11	.11 (.01)	.31 (.10)	.11	.89
High	.10	.13 (.02)	.29 (.08)	.17	.83
RC					
Low	.54	.43 (.18)	.60 (.36)	.34	.66
Average	.36	.16 (.03)	.58 (.33)	.07	.93
High	.34	.21 (.04)	.54 (.30)	.13	.87
WJ III Secondary					
BRS					
Low	.32	.36 (.13)	.44 (.19)	.41	.60
Average	.18	.15 (.02)	.40 (.16)	.13	.88
High	.29	.28 (.08)	.46 (.21)	.27	.73
RC					
Low	.61	.55 (.30)	.55 (.31)	.50	.50
Average	.22	.22 (.05)	.41 (.17)	.22	.78
High	.33	.28 (.08)	.50 (.25)	.24	.76
WJ IV Elementary					
BRS					
Low	.31	.36 (.13)	.42 (.18)	.42	.58
Average	.22	.21 (.04)	.42 (.18)	.20	.80
High	.21	.25 (.06)	.38 (.15)	.30	.70
RC					
Low	.68	.39 (.15)	.73 (.53)	.22	.78
Average	.49	.19 (.04)	.67 (.45)	.07	.93
High	.38	.26 (.07)	.56 (.31)	.18	.82
WJ IV Secondary					
BRS					
Low	.33	.35 (.12)	.46 (.21)	.37	.63
Average	.13	.15 (.02)	.33 (.11)	.17	.83
High	.17	.21 (.04)	.35 (.13)	.26	.74
RC					
Low	.51	.39 (.15)	.60 (.36)	.30	.70
Average	.37	.18 (.03)	.58 (.34)	.09	.91
High	.44	.22 (.05)	.63 (.39)	.11	.89

Note. BRS = Basic Reading Skills, RC = Reading Comprehension.

## Data Availability

Not applicable.
